# PROTOCOL: Electronic mentoring to promote positive youth outcomes for young people under 25: a systematic review

**DOI:** 10.1002/CL2.197

**Published:** 2018-10-16

**Authors:** Robyn M. O'Connor, David L. DuBois, Lucy Bowes

## 1. BACKGROUND FOR THE REVIEW

### 1.1. Description of the problem

Youth unemployment refers to young people between the ages of 15 and 24 years who are without work, but are currently available for and actively seeking work (Youth Employment Network, 2011). Despite a mild recovery between 2009 and 2014, where the number of unemployed youth dropped by 3.3 million, finding employment is still an uphill struggle for 73.3 million youth around the world (International Labour Organization, 2015). In the European Union specifically, more than one in three unemployed youth have been looking for work for more than one year.

Youth who are under‐skilled and under‐educated, who are female, and/or have disabilities are among the groups most at risk of unemployment (International Labour Organization, 2013; World Health Organization & the World Bank, 2011). According to the International Labour Organization (2015), it is therefore crucial that we provide young people with more opportunities to transition to a decent job. This could include: investing in education and training; providing youth with skills that match labour market demands; and social support that levels the playing field so that all aspiring youth can attain productive employment regardless of their gender or other demographic features.

Youth unemployment has been described as causing “scarring effects” (ILO, 2013, p. 2). This means that, unresolved, millions of young people will be at risk of long‐term negative outcomes in relation to their future earnings, employment prospects, health, happiness, and job satisfaction (Bell & Blanchflower, 2011; ILO, 2013; Morsy, 2012). Youth unemployment is also associated with poverty, social exclusion, juvenile delinquency, and distrust in political and welfare systems (Morsy, 2012) – making it a problem faced by the community as a whole, and not just the individual.

Mentoring schemes are widely used to facilitate school‐to‐work transitions. In recent years, there have been an estimated 5,000 mentoring programmes running across the United States serving around three million youth (DuBois, Portillo, et al., 2011). Youth mentoring schemes also form a substantial proportion of the 3,500 mentoring programmes known to be operating in the United Kingdom (Meier, 2008). Despite this, a substantial gap exists between the demand of youth needing or wanting mentors, and the supply of mentors and accessibility of face‐to‐face mentoring programmes (Bruce & Bridgeland, 2014). Increasingly, practitioners have been using electronic mentoring (e‐mentoring) programmes to close the gap (Shpigelman, 2014), but this type of intervention has not yet been rigorously evaluated.

### 1.2. Description of the intervention

#### Context

The first large scale e‐mentoring programme – the ‘Electronic Emissary Project’ – was founded in 1993, enabling students and teachers to be mentored by subject‐matter experts primarily via e‐mail (Shpigelman, 2014, p. 260; Single & Single, 2005, p. 303). Reflecting our increasing reliance on digital technologies, it is perhaps unsurprising that e‐mentoring (also known as online mentoring, virtual mentoring, and telementoring) has emerged as a popular intervention strategy over the last decade in particular.

In 2015, 88% of the population in North America and 74% of the population in Europe were Internet users (Internet World Statistics, 2015). In Great Britain, around 8 in 10 adults (78%) accessed the internet every day or almost every day in 2015 – representing a significant rise compared to 2006, where just over 3 in 10 adults (35%) did so (ONS, 2015). Among young people (aged 16 to 24) specifically, 96% used the Internet ‘on the go’ using a mobile phone, portable computer or handheld device (ONS, 2015).

#### Components

Definitions of ‘e‐mentoring’ vary (e.g. Bierema & Merriam, 2002, p.214; Hamilton & Scandura, 2003, p.388; Single & Muller, 2001, p.108), and may evolve over time with technological advances. According to Shpigelman (2014, p.259), ‘e‐mentoring’ refers to “a relationship in which a mentor, usually a more experienced or an older person, provides guidance and support to a less experienced or younger person (the mentee) via distance communication technologies”.

‘E‐mentoring’ is most commonly done on a one‐to‐one basis, but can also involve more than one mentor supporting the same young person, or one or more mentors supporting a group of young people (Hamilton & Scandura, 2003, p. 397). Formal e‐mentoring relationships established and planned through organisations, institutions, programmes and projects are distinguished from informal or natural mentoring relationships that arise “spontaneously” (Baker & Maguire, 2005, p.15).

More often, e‐mentoring programmes seek to impact vocational and/or educational outcomes (Shpigelman, 2014). For example, MentorNet is a non‐profit e‐mentoring organisation in the US that seeks to increase the entry of underrepresented groups in Science, Technology, Engineering and Maths (STEM) professions. According to the MentorNet website, the organisation has matched over 32,000 STEM students with industry professionals since 1997. In the UK, the Brightside Trust has supported over 45,000 young people since 2003, by linking students with mentors who are undergraduates or employers, to support their transitions into higher/further education and/or employment.

Mentors are usually volunteers. They should not be familially related to the mentee, or mentor as part of their professional or caregiver role; this distinguishes mentoring from professional‐client, parent‐child, and teacher‐student relationships (Tolan, Henry, Schoeny, & Bass, 2008). The amount of support provided by the mentor can vary from one‐to‐two e‐mails or 15 minutes per week, to several hours a week reviewing the mentee's work (Cravens, 2003). Relationship length can be short‐term (under 3 months) to long‐term (an academic or full calendar year) (Shpigelman, 2014).

Whilst traditional mentoring relationships are “nurtured by frequent face‐to‐face contact” (Hamilton & Scandura, 2003, p. 388), e‐mentoring takes place at least partially via computer‐mediated communication (CMC). CMC was originally defined as “a form of electronic written communication” (Shpigelman, 2014, p. 259) and was *asynchronous*‐ that is, communication “not dependent on the physical presence” of users (p. 260). This includes, for example, e‐mail, discussion boards, and web forums. Messages may not be sequential, and users can respond at different times. With technological advancements, e‐mentoring now includes *synchronous* text‐based communication, such as instant messaging and chat rooms where “the users are present and respond in real time” (p. 260), as well as multimedia communication such as Skype and FaceTime, which use audio and/or video tools (e.g., microphones, webcams).

### 1.3. How the intervention might work

#### Logic model

Successful mentoring relationships are usually characterised by a strong and meaningful personal connection forged between the young person and the mentor. According to Rhodes' (2005) widely acknowledged model of youth mentoring, this foundation is posited to catalyse socio‐emotional, cognitive and identity development, which over time leads to positive youth outcomes. Whilst traditional mentoring takes place in person, face‐to‐face, the majority of support provided by an e‐mentor takes place electronically using distance communication technologies. It is therefore necessary to understand how these types of relationships may work differently.

According to Shpigelman (2014), before the process starts, the young person (mentee) needs to have the intrinsic motivation to participate in the programme or receive encouragement from others. Both the mentor and mentee will need to be literate in digital technologies, with adequate reading and writing skills.

Participants must then have access to the appropriate hardware and software, such as a computer, laptop, tablet, or smartphone. This may be through personal ownership, the young person's educational establishment, a library, or an Internet café, for example. Alongside Internet access, participants may also need an e‐mail, instant messaging, or Skype account (depending on the programme). Some e‐mentoring schemes use secure online platforms to encrypt users' e‐mail addresses and monitor messages.

The mentoring itself is characterised by a series of interactions, including trust building, self‐disclosure, empathy and online communication. The mentor may provide informational support (e.g., career advice), tangible assistance (e.g., help with job searching), social and/or emotional support, which may relate directly or indirectly to issues concerning the mentee (p. 264–265). The benefits of receiving this support may be moderated by participants' demographics (e.g., age, gender, race), personalities (e.g., shyness), health (e.g., complexity of any disability), and environmental factors (e.g., family, friends, school) (Shpigelman, 2014, p.264; Shpigelman et al., 2009a, p. 306). Towards the end of the relationship, mentees are expected to reflect upon the information and support gained, so that the results may be valuable beyond the programme (Shpigelman, 2014, p. 265).

It is posited that this process can result in the mentee acquiring or strengthening “the necessary psychological resources” such as “self‐efficacy” and “self‐esteem”, which “will enable goal achievement” (p. 265). Goal achievement may relate to educational, vocational or other positive youth developmental outcomes.

#### Opportunities and challenges

The way in which e‐mentoring programmes work, and their reliance on digital technologies, has been associated with a range of opportunities and challenges (Ensher et al., 2003).

On the one hand, research suggests that the use of electronic communication can make establishing and sustaining relationships easier, and increase the pool of available mentors. This is because the mentor and mentee do not need to be co‐located, and interactions can fit more easily around personal schedules – as the relationship is not dependent on the participants sharing time or space (Bierema & Merriam, 2002, p.219–220; Ensher et al., 2003, p.281). In turn, this can reduce some of the travel and time costs associated with face‐to‐face meetings and written records of interactions may make it easier to check off goals, keep notes of advice, and evaluate mentoring processes (Rhodes, 2003, p. 4).

Demographic features, such as age, race, and gender may be less visible (Bierema & Merriam, 2002), especially in e‐mail only relationships, allowing commonalities and shared interests to be built upon more immediately (Ensher et al., 2003, p. 281). This could be particularly beneficial for individuals with disabilities, women, and ethnic minorities (Ensher et al., 2003, p.282; Hamilton & Scandura, 2003, p. 388). For example, in a post‐evaluation questionnaire of youth and mentors with special needs and/or disabilities who participated in an e‐mentoring programme, some felt virtual communication acted as “a shield” with “protective capacities”, distinct from what may be experienced face‐to‐face (Shpigelman, Weiss, & Reiter, 2009b, p. 923). Even if ‘weak ties’ are built electronically, this may be valuable for disabled individuals who tend to have few relationships outside of their family (Shpigelman et al., 2009a, p. 303), and for whom meeting face‐to‐face may be impractical. Whilst rare, the ability to form ‘idealised’ impressions of each other based on one's own perceptions, rather than what is visible face‐to‐face, may result in ‘hyper‐personal’ relationships (Ensher et al., 2003, p. 277–278).

On the other hand, some research suggests that the mode of communication in e‐mentoring schemes, particularly those that do not use audio or video tools, can be “cold” and impersonal, as participants cannot read body language or hear tone of voice (Ensher et al., 2003, p. 276). Whilst emotions can be imitated through punctuation, jargon such as ‘lol’ (‘laugh‐out‐loud), and emoticons (e.g., ☺ ☹) (Shpigelman, 2009; Shpigelman et al., 2009a), these features may not always add clarity and can be misinterpreted. Additionally, the increased anonymity of some e‐mentoring relationships may increase the likelihood of writing or saying things one would feel to inhibited to communicate face‐to‐face, in‐person. This could result in misunderstandings turning “hostile”, or “flaming” whereby responses are “emotionally charged” (Ensher et al., 2003, p. 276–277).

Online text‐based relationships may be the slowest to develop because of potential time lags between messages (p. 277), and a lack of physical presence and direct observations may restrict role modeling. In the post‐evaluation questionnaire discussed above (Shpigelman et al., 2009b), participants also referred to a “virtual wall”, which they felt acted as “a barrier” or had “disruptive properties” in forming meaningful e‐mentoring relationships (p. 923). It is also too simplistic to say that e‐mentoring programmes are more inclusive by giving less attention to demographic characteristics. For example, the need for digital, Internet, reading and writing skills may restrict accessibility and risk widening the ‘digital divide’. Moreover, in a survey of 1,013 participants in the MentorNet e‐mail mentoring programme, many felt having a mentor of their own race or gender was still important; students who had such reported receiving more help and increased relationship satisfaction (Blake‐Beard, Bayne, Crosby, & Muller, 2011).

Lastly, whilst e‐mentoring schemes omit some of the costs incurred with face‐to‐face mentoring (e.g. in relation to travel expenses), they still require investment in digital technologies and IT resources (Single & Single, 2005, p. 315). Technical issues may cut‐off or disrupt mentor‐mentee communication (Single & Single, 2005, p. 304), and participants may be less willing to share mistakes or career mishaps for fear of privacy and confidentiality issues around online records (Ensher et al., 2003, p. 279).

### 1.4. Why it is important to do this review

#### Existing research on traditional youth mentoring

Traditional, face‐to‐face youth mentoring has been rigorously evaluated. Generally, systematic reviews and meta‐analyses have found this form of mentoring to have small positive effects for young people, for example: an overall effect of *g*=0.21 across behavioural, social, emotional, and academic domains (DuBois et al., 2011); an overall effect of *d*=0.18, with a range of *d*=0.10 for emotional/psychological outcomes to *d*=0.22 for career/employment outcomes (DuBois, Holloway, Valentine, & Cooper, 2002).

However, some have found non‐significant or adverse effects. In a systematic review and meta‐analysis of adolescent school‐based mentoring (Wood and Mayo‐Wilson, 2012), the magnitude of effects across all outcomes were assessed as clinically unimportant, with the largest close to zero: *g*=0.09 for self‐esteem. In Grossman & Rhodes' (2002) re‐analysis of data from a study of 1,138 adolescents randomised to the ‘Big Brothers Big Sisters' mentoring programme or a control condition, high‐risk adolescents with mentor matches terminating within three months suffered significant declines in self‐worth (*p*≤.01) and perceived scholastic competence (*p*≤.05). This suggests that it is important to consider how the length of the mentor‐mentee relationship mediates the outcomes measured.

Some reviews, furthermore, have excluded studies of youth and mentors with ‘major’ disabilities (e.g., Eby, Allen, Evans, Ng, & DuBois, 2008). Including participants with these characteristics, however, is crucial to understanding whether the effects of certain types of mentoring programmes can be generalised to disabled and non‐disabled persons.

#### Existing research on e‐mentoring programmes

Evidence on the effectiveness of e‐mentoring programmes for young people is much less clear. Relative to traditional mentoring projects, these types of interventions have not been in use for as long, and therefore have not had as much time to build a solid evidence base. In the absence of rigorous research, such as systematic reviews, most discussions around e‐mentoring have therefore been speculative or theory‐based (Shpigelman, 2014).

Emerging empirical evidence on e‐mentoring programmes suggests that greater programme satisfaction is associated with frequent third party ‘coaching’ and mandatory pre‐programme mentee training. Single, Muller, and Carlsen (2000) randomly assigned student‐professional e‐mentoring dyads to receive weekly coaching messages or coaching messages every other week, to encourage frequent mentee‐mentor communication and prompt discussion starters. More frequent coaching was associated with greater programme satisfaction. In another study, 400 college/university students (mentees) were randomly assigned to a mandatory or voluntary e‐training group; where training was mandatory, mentors (company professionals) were more satisfied and felt mentees were more engaged (Kasprisin, Single, Single, Ferrier, & Muller, 2008).

Other research has started to construct a body of knowledge on the history and development of e‐mentoring relationships, programme features and associated outcomes, and guidelines for ‘successful’ practice. From 1996 to 2001, the ‘Virtual Volunteering Project’ attempted to index and summarise all known online mentoring programmes (Cravens, 2003). Many developers and coordinators had little or no experience of working with people online, and most were unaware of the research on traditional mentoring (p. 90). Having a facilitator to match participants and review programme goals was concluded to be “critical” to “sustainable” e‐mentoring programmes (p. 91). Between 2000 and 2002, the ‘Digital Heroes Campaign’ matched nearly 250 youth with adults in text‐based e‐mentoring relationships and found that only half lasted the six‐month minimum commitment period or longer (Rhodes, 2003, p. 2), which may be concerning given prematurely terminated short‐term relationships may have iatrogenic effects (Grossman & Rhodes, 2002; Rhodes, 2003, p. 2).

More recently, Lindsay, Hartman & Fellin (2015) conducted a systematic review of mentorship programmes to facilitate transition to post‐secondary education and employment among young people aged 30 or under. The most beneficial programmes were often structured, delivered in group‐based or mixed formats, and lasted longer than 6 months. Interestingly, two of the mentoring interventions identified were conducted online over a two‐ or three‐year period. This shows that studies testing the effectiveness of online mentoring do exist, and suggests that a tailored search strategy, focusing on e‐mentoring specifically, is needed to uncover all of the available evidence on these programmes.

#### What this review will add

Recommendations have been made to governments to consider programmes *proven effective* in facilitating school‐to‐work transitions through providing career guidance to students, in part to address the youth unemployment crisis (ILO, 2012, p. 62, p. 64). Systematic reviews top the hierarchy of evidence for evaluating intervention effectiveness (Sackett, Richardson, Rosenberg, & Haynes, 1997). Whilst there are many traditional mentoring programmes, there is a gap between the supply of and demand for mentors (Bruce & Bridgeland, 2014, p. 2). According to the website of one leading organisation, MentorNet estimates that with funding they could provide e‐mentors to one million young people by 2020. However, organisations and practitioners need to know whether e‐mentoring programmes for youth are a worthwhile investment, and importantly whether there may be any risk of harm.

## 2. OBJECTIVES OF THE REVIEW

The main objective of this review is to answer the question: ‘does electronic mentoring improve psychological, educational, and employment outcomes among young people aged 25 or under?’

## 3. METHODS OF THE REVIEW

### 3.1. Criteria for considering studies for this review

To be included, studies have to meet the following criteria relating to study designs, participants, intervention, comparators, and outcome measures.

#### Study designs

Previous systematic reviews and meta‐analyses on youth mentoring include both experimental and quasi‐experimental designs (e.g., DuBois, Portillo, et al., 2011; Wood & Mayo‐Wilson, 2012), and systematic reviews of the ‘best available’ evidence can be considered more helpful to policy makers and practitioners (Petticrew & Roberts, 2006, p.65).

Consequently, this review will include randomised or quasi‐experimental designs with a prospectively assigned, contemporaneous control group. Without a control group, we cannot begin to attribute any changes in outcomes to the presence or absence of the intervention.

Searches will be limited to studies reported since 1993, when the first large‐scale e‐mentoring programme was founded, and will be conducted in English. For a comprehensive review, studies do not have to appear in peer‐reviewed journals. This means that, for example, PhD dissertations can be included. Books and conference proceedings will not be included.

#### Types of participants

Studies will be eligible if the majority of participants (in the intervention and comparison groups) were young people under the age of 25. This will be ascertained by looking at the age range of participants or, in the absence of that, the mean age of each group – which should be younger than 25 years. Whilst this covers many developmental stages, we anticipate a low number of studies, and therefore think this review would be most valuable in scoping out all of the potentially relevant evidence in this field

Youth and mentors with disabilities and/or special needs will be included in the interests of equity in research. The definition and measurement of disabilities and special needs are not consistent across cultures and institutions. This review will use the definition of disabilities and special needs‐ “physical, emotional, behavioural, or intellectual impairments”‐ as provided by Shpigelman et al. (2009a, p. 305).

#### Type of intervention

To be eligible for this review, studies should evaluate the effectiveness (or efficacy) of an electronic mentoring programme. We will use the following definition to guide our determination of whether the intervention evaluated in any given study constituted an e‐mentoring programme:

*A formal programme or intervention that is intended to promote positive youth outcomes via relationships between young persons (aged under 25) and more experienced or older non‐parental adults or peers, who are acting in a non‐professional helping capacity. All or the majority of mentoring interactions (i.e., more than 50%) must be conducted online (e.g., using e‐mail, instant messaging, Skype), but may be supplemented by telephone calls and face‐to‐face interactions.*

This is distinguished from programmes where the majority of mentoring is done in person, yet the relationship is supplemented via electronic communications, which are not eligible.

This builds on existing, much‐cited definitions used by DuBois et al. (2011) and Ensher et al., (2003). Importantly, if the intervention meets all of the criteria described above, it will be classified as an ‘e‐mentoring’ programme, whether or not the terms ‘e‐mentoring’, ‘mentor’, or ‘mentee’ were used. Programmes can include input from a third party, such as a programme coordinator, to monitor communications and/or send prompts for discussion starters for example.

Interventions that aim to prevent or reduce bullying are not eligible, as they are often fundamentally different from programmes that focus on improving educational or vocational outcomes.

#### Types of comparators

Studies should have at least one control group that received: (1) no formal mentoring programme; or (2) received a formal, traditional face‐to‐face mentoring programme.

#### Types of outcome measures

Studies should report data for a youth outcome in one or more of the following broad categories:

- Psychological/emotional: e.g., self‐esteem, self‐efficacy, confidence
- Academic/school: e.g., standardised test scores, attendance, drop‐out
- Attitudinal/motivational: e.g., career certainty/indecision
- Career/employment: e.g., job interviews, job offers, employment status

This follows the same outcome groupings of a highly cited systematic review in this area (DuBois et al., 2011). Objective and subjective measurements will be eligible, but should use validated and/or published scales where available.

#### Types of adverse effects

The existing literature discussed in the background to this review highlights several potential challenges of e‐mentoring schemes. We will therefore discuss any such negative findings reported in any of the included studies.

#### Types of moderators and mediators

If a sufficient number of studies are identified, we will analyse the following moderators and mediators:

*Participant characteristics*

- Mentee age
- Mentee gender
- Mentee disabled/not‐disabled
- Mentor gender
- Mentor disabled/not‐disabled

*Intervention characteristics*

- Main type of electronic communication used (synchronous vs. asynchronous; text‐based vs. audio/visual)
- Length of relationship (<3 months; 3 to < 6 months; 6 to < 12 months; 12 months or longer)

*Study characteristics*

- Type of control group (no mentoring; face‐to‐face mentoring)
- Study quality (RCT; quasi‐experimental study using propensity score matching; quasi‐experimental study with no matching)

### 3.2. Search methods for identification of studies

This investigation will involve a thorough, highly sensitive search for published and unpublished studies completed or in progress. The search strategy includes multiple electronic databases and international outreach to professional networks.

#### Electronic databases

One reviewer will conduct a systematic literature search of the following 14 electronic databases:

- Applied Social Sciences Index and Abstracts (ASSIA),
- Australian Education Index,
- British Education Index,
- Business Source Complete
- Cochrane Central Register of Controlled Trials in the Cochrane Library,
- Computer and Information Systems Abstracts
- EconLit
- Educational Resources Information Center (ERIC),
- Medline
- PsycINFO (Ovid platform),
- Scopus Social Sciences & Humanities,
- Sociological Abstracts and
- Open Grey, and
- ProQuest Dissertations & Theses

In four of the larger databases (Medline, PsycINFO, ProQuest Dissertations & Theses, and Scopus Social Sciences & Humanities), the terms listed below will be searched for in the title, abstract, and keywords fields. Searches will be restricted to studies from 1993 onwards, and will be conducted in English.

#### Search terms

To maximise sensitivity, we plan to use the following search terms for all 14 electronic databases (adapting proximity operators and wildcards as appropriate due to variations between the databases themselves):

**1 AND (2 OR 3) AND 4**

1. (youth* or teen* or boy* or girl* or adolesc* or minor* or mentee* or protégé* or “young person*” or “young people*” or “young adult*” or student* or pupil* or freshm*n* or undergraduate* or graduate* or juvenile* or sophomore* or junior* or senior*) AND
2. ((e‐mentor* or ementor* or electronic‐mentor* or imentor* or telementor* or cybermentor* or cyber‐mentor*) OR
3. ((electronic* or e‐mail* or email* or “electronic mail*” or “instant messaging” or virtual* or internet or online or cyber or “web‐based” or web* or CMC or computer* or skype or FaceTime or video or e‐communication* or smartphone or laptop or tablet or ipad or telephone or “text message” or Facebook or “asynchronous communication” or “synchronous communication”) ADJ (mentor* or coach* or “non‐parental adult*” or “nonparental adult*” or peer* or “big sister*” or “big brother*” or advis*r*))) AND
4. (control* OR random* OR trial* OR effectiveness OR efficacy OR compar* OR clinical* OR experiment* OR RCT OR QED OR quasi‐experiment* OR pilot OR impact ADJ evaluation OR impact ADJ study OR impact ADJ assessment OR outcome ADJ evaluation OR outcome ADJ study OR outcome ADJ assessment)

#### Searching other resources

- We will conduct an online web search of Google Scholar using key terms such as ‘e‐mentoring’ and ‘online mentoring’ to attempt to identify studies not picked up in our electronic database search. At a minimum, one reviewer will analyse the first 100 results for inclusion.
- One reviewer will also browse the websites of governmental, international, and/or mentoring organisations, such as the UN, to locate other research reports.
- We will contact experts in the field of youth mentoring, to ask them to refer us to any known studies of e‐mentoring programmes potentially relevant to this review.
- One reviewer will hand‐search the reference lists of included studies, and key previous reviews in this area.

### 3.3. Description of methods used in primary research

The Campbell Collaboration recommends including a brief description of the methods used in at least one primary research study that might be eligible for inclusion.

Smith‐Jentsch, Scielzo, Yarbrough, & Rosopa (2008) conducted a randomised controlled efficacy trial in a university campus in the USA. 106 participants were randomly assigned to receive the e‐mentoring programme (intervention group), or face‐to‐face mentoring (control group). The programme was designed to ease their transition from high school to university life. All communication in the intervention group was synchronous via private computer‐based chat‐rooms, 15 minutes once a week for three weeks. Mentors were paid $10 per hour for completing measures. The researchers used The College Self‐Efficacy Inventory’ to measure participants' self‐efficacy, in terms of how confident they felt in performing 15 different course and social‐related tasks on a six‐point scale. Students completed surveys prior to randomisation, and again following the end of the intervention.

### 3.4. Data collection and analysis

#### Selection of studies

The reference management software EndNote will be used to merge the electronic search results and remove duplicates. One reviewer will examine all titles and abstracts to remove obviously irrelevant reports, and retrieve the full‐text versions of all potentially relevant reports and reports where eligibility was unclear from their abstract, including through contact with study authors.

One reviewer will assess the full‐text reports for compliance with the eligibility criteria. Studies not meeting the eligibility criteria will be excluded, and studies meeting the eligibility will be included.

A second reviewer will be responsible for confirming a random sample of at least 25% of the excluded studies. Any disagreements will be resolved through consensus with the third reviewer.

A second reviewer will be responsible for independently assessing the eligibility of all the included studies. Any disagreements will be resolved through consensus with the third reviewer.

The search process will be outlined in a PRISMA flow diagram (Moher, Liberati, Tetzlaff, Altman, & The PRISMA Group, 2009).

#### Data extraction and management

A data extraction form will be created to extract data from included studies, based on the Cochrane Handbook's checklist of items to consider in data extraction (Higgins & Deeks, 2011), and a data extraction form used in a previous systematic review of youth mentoring (Wood & Mayo‐Wilson, 2012), obtained from the author (S. Wood, personal communication, July 2014).

Two reviewers will independently extract and record data electronically from the included studies, to minimise errors. Any disagreements will be resolved through consensus with the third reviewer.

Characteristics of included studies will be summarised in a table, as well as summaries of findings for each outcome.

#### Assessment of risk of bias in included studies

Two reviewers will use the Cochrane Collaboration's risk of bias tool for randomised controlled studies (Higgins, Altman, & Sterne, 2011). Each will be rated as having high, low, or unclear risks of bias due to sequence generation, allocation concealment, blinding, incomplete data, selective outcome reporting, and other sources if bias. The same tool will be used to assess bias in any included quasi‐experimental studies, but rather than assessing the sequence generation, we will assess any steps taken to match the intervention and control groups. Any disagreements will be resolved through consensus with the third reviewer.

#### Measures of treatment effect

We will report a standardised difference in post‐test means between the intervention and control group for each study. For dichotomous measures, we will calculate odds ratios with a 95% confidence interval. For continuous data, we will express the effect size as a Hedges' *g.* In general, this involves taking the raw difference between treatment and control group means on the outcome measure at post‐test, and then dividing this difference by the pooled (weighted average) standard deviation of the measure for the two groups. This is the method used in two other systematic reviews and meta‐analyses of youth mentoring programmes (DuBois et al., 2011; Wood & Mayo‐Wilson, 2012).

#### Unit of analysis issues

In some studies, effect sizes may be based on observations that are potentially non‐independent due to clustering, such as within classrooms or schools. To avoid unit‐of‐analysis errors, we plan to calculate all effect sizes at the individual level, and to cluster‐adjust any that need it. This will also enable us to conduct a meta‐analysis, should sufficient studies be identified.

If a study compares multiple intervention/control groups, we will follow the Cochrane Collaboration's recommended method of combining all relevant experimental intervention groups of the study into a single group, and combining all relevant control groups into a single control group. We will then calculate the effect size in the usual way.

At a minimum, studies are expected to contribute one effect size for at least one of the outcomes specified. We will allow studies to contribute more than one effect size, where the effects relate to different outcomes (for example, one on school test results, and one on employment status). If a study calculates more than one effect size for the same outcome (for example, attendance rates per school records vs. self‐reported attendance rates at school), then the most objective and/or validated measure will be used.

#### Dealing with missing data

We will contact the authors of any included study with missing data required for our analysis. Whilst methods are available to impute some missing data such as standard deviations, they involve making assumptions about unknown statistics, and following guidance in the Cochrane Handbook will be avoided if missing in the majority of studies (Higgins, Deeks, & Altman, 2011).

#### Data synthesis

Based on the small number of studies that are likely to be included in this review, the authors plan to conduct a narrative summary of the studies and their findings in accordance with the inclusion criteria, including any effect sizes extracted and calculated (by us or the original study authors). Effect sizes will be converted to standardised mean differences as explained in the ‘Measures of treatment effect’ section above.

If sufficient studies are identified and, subject to the considerations below, a meta‐analysis is deemed appropriate, we will use a random‐effects model. Because effect‐size information will be reported for the overall sample in most reports, each report or study generally will contribute one sample to the analysis of a given outcome. For each type of comparison, standardised mean effect sizes with 95% confidence intervals will be reported for each outcome. Effect sizes will be weighted by the inverse of their variances to provide more efficient estimations of true population effects (Hedges & Olkin, 1985). This procedure gives greater weight to effect sizes based on larger samples, and is generally the preferred approach (Cooper, 2010).

We will also test for statistical significance of heterogeneity in effect sizes between studies, using the chi‐squared test. However, we acknowledge that care must be taken in interpreting the *Q* statistic, since it can have low power in a meta‐analysis when studies have small sample size or are few in number (Deeks, Higgins, & Altman, 2011). Additionally, some researchers argue that since clinical and methodological diversity always occur in a meta‐analysis, statistical heterogeneity is inevitable and therefore the test for heterogeneity may be irrelevant to the choice of fixed or random‐effects model analysis (Deeks, Higgins, & Altman, 2011). Indeed, previous reviews have shown that substantial variation exists in mentoring interventions and evaluations (e.g., DuBois et al., 2002, 2011). In view of these considerations, we anticipate conducting all analyses using a random‐effects model regardless of the outcome of significance tests for heterogeneity. However, we will conduct a sensitivity analysis to investigate the robustness of our findings to this decision (using a fixed rather than random effects model).

If the number of available studies is sufficient, potential moderators and mediators of mentoring‐programme effect sizes as described earlier will be investigated. The statistical power of all tests of moderators will be reported (Hedges & Pigott, 2004) and will be required to be at least .50 for detecting effect size differences of .20 or greater in order to proceed with testing.

We aim to minimise publication bias though searching for and including both published and unpublished studies that meet our eligibility criteria. Nevertheless, we will use funnel plots for information about possible publication bias if we find sufficient studies (Sterne, Egger, & Moher, 2011). However, asymmetric funnel plots are not necessarily caused by publication bias (and publication bias does not necessarily cause asymmetry in a funnel plot). If asymmetry is present, we will consider possible reasons for this.

## 4. PRELIMINARY TIMEFRAME

We plan to submit a full draft of the review within six to eight months of the publication of this protocol.

## 5. PLANS TO UPDATE THE REVIEW

The authors assume responsibility for updating the review every three to five years. If we are no longer able to resume this responsibility, we will inform the Campbell Library and ensure an appropriate transfer of responsibility is made.

## 6. POTENTIAL CONFLICTS OF INTEREST

DLD has been involved in prior published reviews of youth mentoring programmes. However, these reviews did not address the effectiveness of CMC‐only e‐mentoring programmes, the focus of this review. RMO is the co‐author of practical guidance, published by the Early Intervention Foundation, on how to choose, commission, and evaluate mentoring services (O’Connor & Waddell, 2015); however, the guidance does not address the effectiveness e‐mentoring programmes, and has a specific focus on preventing gang and youth violence. LB is not aware of any potential conflicts of interest.

All authors will strive to interpret the results without prejudice or bias.

## 7. FUNDING

None.
